# Risk of anastomotic leakage with two-row versus three-row manual circular staplers in colorectal anastomosis: a U.S. cohort study

**DOI:** 10.1007/s00384-023-04552-0

**Published:** 2023-11-07

**Authors:** Tongtong Wang, Mordechai Sadowsky, Rebekah Blakney, Paul Coplan, William Petraiuolo, Mark Soberman, Joerg Tomaszewski, Lexi Rene, Jennifer Wood

**Affiliations:** 1 Epidemiology & Real-World Data Sciences, MedTech, Johnson & Johnson, New Brunswick, NJ USA; 2 Medical Safety, MedTech, Johnson & Johnson, New Brunswick, NJ USA; 3 Medical Affairs, MedTech, Johnson & Johnson, Raritan, NJ USA

**Keywords:** Anastomotic leak, Manual circular staplers, Left-sided colorectal surgery, Retrospective cohort study, Premier Healthcare Database (PHD)

## Abstract

**Purposes:**

To compare the risk of anastomotic leak (AL) between Ethicon manual circular staplers (two-row) versus Medtronic EEA^™^ circular stapler with Tri-Staple^™^ technology (three-row) and between Medtronic EEA^™^ circular stapler with DST^™^ Series technology (two-row) versus Tri-Staple^™^ technology.

**Methods:**

A retrospective cohort study was conducted in adult patients who underwent a left-sided colorectal surgery 2019–2022 in U.S. Premier Healthcare Database to assess the risk of AL within 30 days post-index procedure. The study devices were Ethicon manual circular staplers, Medtronic EEA^™^ circular stapler with DST^™^ technology, and Medtronic EEA^™^ circular stapler with Tri-Staple^™^ technology.

**Results:**

Across 447 hospitals, the cumulative incidences (95% confidence intervals [CI]) of AL within 30 days post-index procedure were 7.78% (6.91–8.74%) among 8337 patients in the Ethicon manual circular stapler cohort, 7.54% (6.87–8.27%) among 7928 patients in the Medtronic EEA^™^ circular stapler with DST^™^ technology cohort, and 8.19% (6.57–10.07%) among 1306 patients in the Medtronic EEA^™^ circular stapler with Tri-Staple^™^ technology cohort. Comparative analyses revealed no difference comparing Ethicon manual circular staplers with Medtronic EEA^™^ circular staplers with Tri-Staple^™^ technology (risk ratio [RR], 0.72; 95% CI, 0.52–1.01) or comparing Medtronic EEA^™^ circular staplers with DST^™^ technology to Tri-Staple^™^ technology (RR, 0.75; 95% CI, 0.53–1.06).

**Conclusion:**

In this analysis of a large cohort of patients undergoing a left-sided colorectal surgery from a U.S. hospital database, the risk of AL observed with manual two-row circular staplers was similar to that seen with three-row devices. This study affirms the safety of manual two-row circular staplers in colorectal anastomosis.

## Introduction

Prevention of anastomotic leak (AL) is an important goal in colorectal anastomosis [1, 2]. AL causes significant morbidity and mortality [3–6]. Several patient-related factors have been identified as risk factors for AL, including male sex, low anterior resection (more distal position of anastomosis), malnutrition, obesity, and diabetes [7–10].

With the development of novel circular stapling devices to facilitate colorectal anastomosis, there has been increased interest in specific device mechanics and their impact on AL risks. The primary variations in the stapling mechanics of commonly used circular staplers include closed height of staples, mechanical vs. powered mechanism for firing staples, shape of final staple form, and number of rows of staples. Various preclinical and clinical studies have demonstrated the safety and efficacy of these devices [11–16], but there are relatively few head-to-head studies of different devices and no prospective trials.

A retrospective single-center study by Mazaki et al. compared the Medtronic EEA^™^ circular stapler with Tri-Staple^™^ technology (a manual three-row circular stapler) to an unspecified two-row circular stapler in 256 patients with left-sided colon cancer resections and found risks of AL in the two-row and three-row groups of 7.7% and 2.7%, respectively (*p* = 0.23) [17]. A subsequent analyses added 29 patients without cancer and found a significantly higher AL risk in the two-row stapling group than the three-row group after propensity score matching (11.6% vs. 1.8%; *p* = 0.04) [18].

In another single-center head-to-head circular stapler comparison, Quero et al. [10] reported a lower overall AL risk after 375 rectal cancer resections in the Medtronic EEA^™^ circular stapler with Tri-Staple^™^ technology group (6/178, 3.4%) than the two-row stapler group (unspecified combination of Medtronic EEA^™^ circular stapler with DST^™^ Series technology and Ethicon^™^ circular stapler, 19/197, 9.6%) (*p* = 0.01). However, the risk of ISREC grade C AL [19], a severe form of AL leading to re-operation, did not differ between the two groups (2.5% in the two-row vs. 2.2% in the three-row circular stapler group).

These single-center retrospective studies not only have the potential for strong confounding due to within-institution treatment selection forces [20] but also can lack generalizability of AL risk due to small sample size and idiosyncratic regional, institutional, and patient characteristics. The Italian ColoRectal Anastomotic Leakage Study analyzed 2799 patients from 78 centers after elective left-sided colorectal resections (5.5% with open surgery) and reported a 5.0% AL risk in the overall study population [21]. After propensity score matching, only 850 patients were retained in the comparative analysis, showing the Medtronic EEA^™^ circular stapler with Tri-Staple^™^ technology (9/425, 2.1%) had a lower AL risk than the two-row stapler group (26/425, 6.1%) (*p* = 0.01). However, surgeon experience and intraoperative anastomotic testing and reinforcement were not measured in the study, which could affect the study results.

A comparative analysis of the risk of AL among two-row versus three-row manual circular staplers in colorectal anastomosis using a nationally representative database has not been conducted in the United States (U.S.). Healthcare databases can be a useful source of data for safety surveillance of medical devices and include a much larger sample size and many more hospitals than possible with single-center studies. Using data from a large U.S. hospital database, we sought to compare the risk of AL among patients who underwent left-sided colorectal surgery with anastomosis using a two-row Ethicon manual circular stapler versus the Medtronic EEA^™^ circular stapler with Tri-Staple^™^ technology. Additionally, the risk of AL among patients treated with the two-row Medtronic EEA^™^ circular stapler with DST^™^ Series technology was separately compared to those treated with the Tri-Staple^™^ technology.

## Methods

### Study design

A retrospective cohort study was conducted using electronic healthcare data from the Premier Healthcare Database (PHD). The PHD is a large, U.S. hospital-based, service-level, all-payer database that contains information on inpatient discharges from geographically diverse hospitals, including nonprofit, nongovernmental, community, and teaching hospitals in both rural and urban areas. More than 1000 hospitals have contributed data, including records for > 10 million visits per year [20]. The PHD consists of de-identified healthcare records. In the U.S., retrospective analyses of PHD data are considered exempt from informed consent and approval by an institutional review board.

### Study population

Patients meeting all of the following criteria were included in the study: those who had billing charges for a study device (Ethicon manual circular staplers, Medtronic EEA^™^ circular stapler with DST Series^™^ technology, or Medtronic EEA^™^ circular stapler with Tri-Staple^™^ technology) at the index admission (i.e., the initial admission where a study device was identified) between January 1, 2019, and November 30, 2022; who underwent a left-sided colorectal surgery (i.e., the index procedure), defined as presence of a qualifying procedure code for left-sided colorectal surgery, at the index admission; and who were ≥ 18 years at the index admission.

Patients were excluded from the study if they had any of the following: missing data on age or sex (an indicator of poor data quality), presence of a diagnosis indicating AL on admission during the index admission, and billing charges for ≥ 2 study devices of interest during the index admission.

### Study devices

The study population was classified by device used: (1) Ethicon manual circular staplers (two-row), (2) Medtronic EEA^™^ circular stapler with DST^™^ technology (two-row), and (3) Medtronic EEA^™^ circular stapler with Tri-Staple^™^ technology (three-row). Ethicon manual circular staplers included Ethicon Legacy Curved Intraluminal Stapler, Legacy Endoscopic Intraluminal Stapler, and Ethicon^™^ Circular Stapler, XL Sealed. The ECHELON CIRCULAR^™^ Powered Stapler, which uses powered mechanism for firing staples, was not included in the study due to the different technology in comparison to manual circular staplers (fired under manual grip force). The identification of devices was based on a query of unstructured text fields in the PHD for model numbers and brand names, including variants such as misspellings and abbreviations.

### Study outcome

The study outcome was AL within 30 days of the index procedure. A 30-day follow-up period was chosen as AL occurs at a median of 12 days (range, 3–30 days) after a colorectal procedure, and it is the standard post-operative follow-up period for most studies assessing AL after stapled anastomosis [22]. As there is no specific diagnosis code in the International Classification of Diseases, 10^th^ Revision, Clinical Modification (ICD-10-CM) to assess AL, the incidence of AL was estimated via surrogate diagnoses that usually occur concomitantly with a leak. These included peritoneal abscess, peritonitis, fistula of intestine, or stoma formation post colorectal anastomosis procedure. As such, the presence of an ICD-10-CM diagnosis code for AL surrogate diagnoses (K63.2, K65.0, K65.1, K91.89, and Y83.2) either during the index admission or during a re-admission within 30 days post-index procedure or the presence of an ICD-10 procedure code indicating a diverting stoma (0D18%%4, 0D1B%%4, 0D1E%%4, 0D1H%%4, 0D1K%%4, 0D1L%%4, 0D1M%%4, 0D1N%%4) occurring within 1–30 days post-index procedure was used to define AL in the study. A similar approach has been used in studies assessing the risk of AL using electronic healthcare data, such as PHD [23] and the Nationwide Inpatient Sample database [24].

### Statistical analyses

Descriptive analyses were performed to summarize the demographic, clinical, procedural, and provider characteristics at the time of the index procedure by study device. Unadjusted cumulative incidences and 95% confidence intervals (CIs) were estimated for all study cohorts and further stratified by key patient and provider characteristics.

We performed two head-to-head comparisons to estimate the risk ratio (RR) and 95% CI of AL for Ethicon manual circular staplers (two-row) compared to Medtronic EEA^™^ circular staplers with Tri-Staple^™^ technology (three-row) and to estimate the RR (95% CI) of AL for the Medtronic EEA^™^ circular stapler with DST^™^ technology compared to Tri-Staple^™^ technology. A propensity score weighting (PSW) method was used to control for potential confounders (i.e., demographic, clinical, procedural, and provider characteristics) by balancing the distribution of baseline characteristics between the comparison groups.

Trimmed and untrimmed PSW methods that estimate the average treatment effect on the treated were implemented and compared on the basis of covariate balance as measured by absolute standardized differences (ASD) in means and proportions, which compares difference in means or proportions in pooled standard deviation units. The PSW trimmed at the 99^th^ percentile performed the best as measured by the fewest number of variables imbalanced (as indicated by an ASD value > 0.10) and the lowest mean ASD across all balanced covariates. One data analyst balanced the data without access to the outcome data and another data analyst performed the outcome analysis using the balanced data, thus removing the potential for bias resulting from repeated applications of covariate balancing to obtain a desired study outcome [25–27].

In the final PSW cohorts, covariate-balanced cumulative incidences and 95% CIs were estimated for all device cohorts. A weighted (covariate balance weights) log-binomial regression model was used with treatment as the only explanatory variable to estimate the covariate balanced RR (target group versus the comparator group for the study outcome of interest). A cluster (hospital) robust standard error approach was applied to estimate the variability in the RR estimate and to construct two-sided 95% CIs.

With the sample size available for the analysis (8000 in the 2-row manual circular stapler cohort and 1300 in the 3-row manual circular stapler cohort) and a cumulative incidence of AL of 8% in the 3-row manual circular stapler cohort, a power analysis indicated power of 0.995 (99.5%) to detect a RR of 1.5 or above in a 2-row manual circular stapler relative to a 3-row manual circular stapler at a significance level of 0.05 (or type I error of 0.05) in a two-sided test.

To evaluate the impact of an elective diverting stoma on the AL risk estimate, a secondary analysis was conducted to assess the association between the use of a two- vs. three-row circular stapler and the risk of AL among those who did not have a diverting stoma procedure prior to or on the same day as the index procedure. To minimize the potential impact of outcome misclassification resulting from loss of continuous enrollment in the PHD, a sensitivity analysis was conducted in patients from hospitals that had ≥ 30 days continuous enrollment in PHD after the patients’ index procedure.

## Results

### Study population

Across 447 U.S. hospitals, we identified 8337 patients who underwent a left-sided colorectal surgery with the use of Ethicon manual circular staplers, 7928 with the use of the Medtronic EEA^™^ circular stapler with DST^™^ technology, and 1306 with the use of Medtronic EEA^™^ circular stapler with Tri-Staple^™^ technology between January 1, 2019, and November 30, 2022. Most patients were white (80%), female (54%), and had a mean (SD) age of 60 (13) years. The majority of patients (64%) underwent a sigmoid colectomy based on the primary procedure code. The index procedures were predominantly performed in an inpatient setting (99%), elective (nonurgent, 84%), and in a large hospital (76%). Over 40% of patients in the two-row circular stapler cohorts underwent an open surgery, while 40% in the three-row cohort underwent a robotic-assisted surgery. Approximately 16% had a diverting stoma prior to or on the same day as the index procedure during the index admission, which was similar across the three cohorts. Among those, 99% had a diverting stoma identified on the same day as the index procedure (Table 1).

**Table 1:** Patient characteristics at the index admission by study device

	**Ethicon manual circular staplers**		**Medtronic EEA**^**™**^** circular stapler with DST Series**^**™**^** technology**		**Medtronic EEA**^**™**^** circular stapler with Tri-Staple**^**™**^** technology**	
	***n***	**%**	***n***	**%**	***n***	**%**
*N*	8337		7928		1306	
Sex: male	3848	46.2%	3733	47.1%	584	44.7%
Age						
Mean (standard deviation)	60.2 (13.5)		59.8 (13.6)		60.6 (12.9)	
18–< 45 years	1098	13.2%	1059	13.4%	153	11.7%
45–< 65 years	3872	46.4%	3742	47.2%	624	47.8%
65–< 75 years	2132	25.6%	2046	25.8%	353	27.0%
75 years or older	1235	14.8%	1081	13.6%	176	13.5%
Race						
White	6892	82.7%	6036	76.1%	1131	86.6%
Black	603	7.2%	648	8.2%	90	6.9%
Others	707	8.5%	1105	13.9%	62	4.8%
Unspecified	135	1.6%	139	1.8%	23	1.8%
Procedure year						
2019	3317	39.8%	3215	40.6%	293	22.4%
2020	2258	27.1%	2052	25.9%	282	21.6%
2021	1803	21.6%	1761	22.2%	379	29.0%
2022, through Nov 30 2022	959	11.5%	900	11.4%	352	27.0%
Surgical site based on primary procedure code						
Rectum	670	8.0%	763	9.6%	166	12.7%
Sigmoid	5395	64.7%	5043	63.6%	838	64.2%
Descending colon	401	4.8%	305	3.9%	55	4.2%
Others	1871	22.4%	1817	22.9%	247	18.9%
Primary diagnosis						
Malignant neoplasms	2099	25.2%	2358	30.0%	358	27.4%
Benign neoplasm	178	2.1%	141	1.8%	19	1.5%
Diverticular disease or Diverticulitis	3477	41.7%	3050	38.5%	601	46.0%
Intestinal obstruction	243	2.9%	240	3.0%	34	2.6%
Others	2340	28.1%	2139	27.0%	294	22.5%
Surgical approach						
Open	3566	42.8%	3328	42.0%	409	31.3%
Laparoscopic	2555	30.7%	2732	34.5%	375	28.7%
Robotic assisted	2216	26.6%	1868	23.6%	522	40.0%
Admission type: elective	6894	82.7%	6780	85.5%	1141	87.4%
Diverting stoma occurred prior to or on the same day as the index procedure	1355	16.3%	1350	17.0%	224	17.2%
Surgeon specialty						
General surgeon	4549	54.6%	4369	55.1%	477	36.5%
Colon/rectal surgeon	2784	33.4%	2484	31.3%	647	49.5%
Others or unspecified	1004	12.0%	1075	13.6%	182	14.0%
Charlson Comorbidity Index scores						
0	3488	41.8%	3106	39.2%	517	39.6%
1–2	2908	34.9%	2838	35.8%	469	35.9%
3–4	965	11.6%	917	11.6%	164	12.6%
5+	976	11.7%	1067	13.5%	156	11.9%
Cardiovascular diseases	1295	15.5%	1147	14.5%	205	15.7%
Chronic obstructive pulmonary disease	599	7.2%	573	7.2%	118	9.0%
Coagulation defects	101	1.2%	71	0.9%	13	1.0%
Diabetes	1436	17.2%	1342	16.9%	256	19.6%
Hypertension	4407	52.9%	4108	51.8%	723	55.4%
Immunodeficiency/immunosuppression	115	1.4%	135	1.7%	19	1.5%
Kidney disease	601	7.2%	502	6.3%	91	7.0%
Malnutrition	397	4.8%	437	5.5%	59	4.5%
Obesity	1885	22.6%	1759	22.2%	334	25.6%
Hospital setting: inpatient	8334	99.96%	7806	98.5%	1305	99.9%
Location: urban hospital	7501	90.0%	7037	88.8%	1061	81.2%
Teaching hospital	3757	45.1%	4515	57.0%	668	51.2%
Hospital region						
Midwest	1208	14.5%	1848	23.3%	328	25.1%
Northeast	1710	20.5%	1777	22.4%	58	4.4%
South	4398	52.8%	3245	41.0%	755	57.8%
West	1021	12.3%	1058	13.4%	165	12.6%
Hospital size						
Large	6157	73.9%	6253	78.9%	1027	78.6%
Medium	1668	20.0%	1126	14.2%	220	16.9%
Small	512	6.1%	549	6.9%	59	4.5%

Unadjusted cumulative incidences of AL (95% CIs) were 7.78% (6.91–8.74%) in the Ethicon manual circular stapler cohort, 7.54% (6.87–8.27%) in the Medtronic EEA^™^ circular stapler with DST^™^ technology cohort, and 8.19% (6.57–10.07%) in the Medtronic EEA^™^ circular stapler with Tri-Staple^™^ technology cohort within 30 days post-index procedure (Tables 2 and 3).

**Table 2:** Risk estimates for anastomotic leak—Ethicon manual circular staplers vs. Medtronic EEA^™^ circular stapler with Tri-Staple^™^ technology

	**Number of patients**^**b**^	**Number of events**	**Cumulative incidence (95% confidence interval)**	**Risk ratio (95% confidence interval)**
**Primary analysis**				
**Before propensity score weighting**				
Ethicon manual circular staplers	8337	649	7.78% (6.91–8.74%)	0.95 (0.75–1.20)
Medtronic EEA^™^ circular stapler with Tri-Staple^™^ technology	1306	107	8.19% (6.57–10.07%)	
**After propensity score weighting**^**a**^				
Ethicon manual circular staplers	8337	649	7.78% (6.91–8.74%)	0.72 (0.52–1.01)
Medtronic EEA^™^ circular stapler with Tri-Staple^™^ technology	7419	801	10.79% (7.6–14.74%)	

**Subgroup analysis (among patients who did not have diverting stoma prior to or at the same day as the index procedure)**				
**Before propensity score weighting**				
Ethicon manual circular staplers	6982	495	7.09% (6.21–8.05%)	0.98 (0.77–1.26)
Medtronic EEA^™^ circular stapler with Tri-Staple^™^ technology	1082	78	7.21% (5.67–9.01%)	
**After propensity score weighting**^**a**^				
Ethicon manual circular staplers	6982	495	7.09% (6.21–8.05%)	0.77 (0.50–1.18)
Medtronic EEA^™^ circular stapler with Tri-Staple^™^ technology	5892	546	9.27% (5.75–13.95%)	

**Sensitivity analysis (institutions with ≥ 30-day continuous enrollment in the PHD)**				
**Before propensity score weighting**				
Ethicon manual circular staplers	8296	646	7.79% (6.90–8.74%)	0.96 (0.77–1.20)
Medtronic EEA^™^ circular stapler with Tri-Staple^™^ technology	1293	105	8.12% (6.58–9.88%)	
**After propensity score weighting**^**a**^				
Ethicon manual circular staplers	8296	646	7.79% (6.90–8.74%)	0.73 (0.52–1.01)
Medtronic EEA^™^ circular stapler with Tri-Staple^™^ technology	7345	788	10.73% (7.58–14.64%)	

^a^The variables included in the propensity score model included patient demographics (age, sex, and race), clinical characteristics (Charlson Comorbidity Index, comorbid conditions, diverting stoma occurred prior to or on the same day as the index procedure), procedural characteristics (procedure year, surgical site, primary diagnosis, surgical approach, and admission type), and hospital and provider characteristics (hospital region, hospital location, hospital setting, hospital bed size, and surgeon specialty)

^b^After propensity score weighting, the number of patients in the Medtronic EEA^™^ circular stapler with Tri-Staple^™^ technology reflects a weighted sample size

**Table 3:** Risk estimates for anastomotic leak—Medtronic EEA^™^ circular stapler with DST Series^™^ technology vs. Tri-Staple^™^ technology

	**Number of patients**^**b**^	**Number of events**	**Cumulative incidence (95% confidence interval)**	**Risk ratio (95% confidence interval)**
**Primary analysis**				
**Before propensity score weighting**				
Medtronic EEA^™^ circular stapler with DST Series^™^ technology	7928	598	7.54% (6.87–8.27%)	0.92 (0.74–1.14)
Medtronic EEA^™^ circular stapler with Tri-Staple^™^ technology	1306	107	8.19% (6.57–10.07%)	
**After propensity score weighting**^**a**^				
Medtronic EEA^™^ circular stapler with DST Series^™^ technology	7928	598	7.54% (6.87–8.27%)	0.75 (0.53–1.06)
Medtronic EEA^™^ circular stapler with Tri-Staple^™^ technology	7086	710	10.03% (6.86–14.02%)	

**Subgroup analysis (among patients who did not have diverting stoma prior to or at the same day as the index procedure)**				
**Before propensity score weighting**				
Medtronic EEA^™^ circular stapler with DST Series^™^ technology	6578	451	6.86% (6.19–7.57%)	0.95 (0.75–1.20)
Medtronic EEA^™^ circular stapler with Tri-Staple^™^ technology	1082	78	7.21% (5.67–9.01%)	
**After propensity score weighting**^**a**^				
Medtronic EEA^™^ circular stapler with DST Series^™^ technology	6578	451	6.86% (6.19–7.57%)	0.80 (0.51–1.26)
Medtronic EEA^™^ circular stapler with Tri-Staple^™^ technology	5678	485	8.54% (5.08–13.28%)	

**Sensitivity analysis (institutions with ≥ 30-day continuous enrollment in the PHD)**				
**Before propensity score weighting**				
Medtronic EEA^™^ circular stapler with DST Series^™^ technology	7885	595	7.55% (6.87–8.27%)	0.93 (0.75–1.14)
Medtronic EEA^™^ circular stapler with Tri-Staple^™^ technology	1293	105	8.12% (6.58–9.88%)	
**After propensity score weighting**^**a**^				
Medtronic EEA^™^ circular stapler with DST Series^™^ technology	7885	595	7.55% (6.87–8.27%)	0.75 (0.53–1.06)
Medtronic EEA^™^ circular stapler with Tri-Staple^™^ technology	7055	708	10.03% (6.86–14.03%)	

^a^The variables included in the propensity score model included patient demographics (age, sex, and race), clinical characteristics (Charlson Comorbidity Index, comorbid conditions, diverting stoma occurred prior to or on the same day as the index procedure), procedural characteristics (procedure year, surgical site, primary diagnosis, surgical approach, and admission type), and hospital and provider characteristics (hospital region, hospital location, hospital setting, hospital bed size, and surgeon specialty)

^b^After propensity score weighting, the number of patients in the Medtronic EEA^™^ circular stapler with Tri-Staple^™^ technology reflects a weighted sample size

### Comparative risk of AL within 30 days post-index procedure

#### Ethicon manual circular staplers vs. Medtronic EEA™ circular stapler with Tri-Staple™ technology

Some imbalances in baseline characteristics between the Ethicon manual circular stapler and Medtronic EEA^™^ circular stapler with Tri-Staple^™^ technology cohorts were observed. Following PSW, adequate covariate balance between two cohorts was achieved (Table 4).

**Table 4:** Patient and hospital characteristics at the index admission after propensity score weighting: Ethicon manual circular staplers vs. Medtronic EEA^™^ circular stapler with Tri-Staple^™^ technology

	**Ethicon manual circular staplers**		**Medtronic EEA**^**™**^** circular stapler with Tri-Staple**^**™**^** technology**		**Absolute standardized difference**
	***n***	**%**	***n***	**%**	
*N*	8337		7419^a^		
Sex: male	3848	46.2%	3573	48.2%	0.040
Age					
18–< 45 years	1098	13.2%	1110	15.0%	0.062
45–< 65 years	3872	46.4%	3256	43.9%	
65–< 75 years	2132	25.6%	1924	25.9%	
75 years or older	1235	14.8%	1129	15.2%	
Race					
White	6892	82.7%	6144	82.8%	0.051
Black	603	7.2%	458	6.2%	
Others	707	8.5%	695	9.4%	
Unknown	135	1.6%	122	1.6%	
Procedure year					
2019	3317	39.8%	2877	38.8%	0.167
2020	2258	27.1%	1610	21.7%	
2021	1803	21.6%	1758	23.7%	
2022, through Nov 2022	959	11.5%	1174	15.8%	
Surgical site					
Rectum	670	8.0%	628	8.5%	0.062
Sigmoid	5395	64.7%	4583	61.8%	
Descending colon	401	4.8%	405	5.5%	
Others	1871	22.4%	1803	24.3%	
Surgical approach					
Open	3566	42.8%	3335	45.0%	0.101
Laparoscopic	2555	30.6%	1938	26.1%	
Robotic assisted	2216	26.6%	2145	28.9%	
Primary diagnosis					
Malignant neoplasms	2099	25.2%	2050	27.6%	0.074
Benign neoplasm	178	2.1%	156	2.1%	
Diverticular disease or diverticulitis	3477	41.7%	2844	38.3%	
Intestinal obstruction	243	2.9%	220	3.0%	
Others	2340	28.1%	2149	29.0%	
Diverting stoma occurred prior to or on the same day as the index procedure	1355	16.3%	1403	18.9%	0.070
Cardiovascular diseases	1295	15.5%	1169	15.8%	0.006
Chronic obstructive pulmonary disease	599	7.2%	552	7.4%	0.010
Coagulation defects	101	1.2%	102	1.4%	0.015
Diabetes	1436	17.2%	1302	17.5%	0.008
Hypertension	4407	52.9%	3872	52.2%	0.013
Immunodeficiency/immunosuppression	115	1.4%	100	1.3%	0.003
Kidney disease	601	7.2%	522	7.0%	0.007
Malnutrition	397	4.8%	341	4.6%	0.008
Obesity	1885	22.6%	1789	24.1%	0.036
Admission type: elective	6894	82.7%	6053	81.6%	0.029
Hospital setting: inpatient	8334	100.0%	7417	100.0%	0.008
Location: urban hospital	7501	90.0%	6734	90.8%	0.027
Teaching hospital	3757	45.1%	3500	47.2%	0.043
Hospital region					
Midwest	1208	14.5%	1223	16.5%	0.154
Northeast	1710	20.5%	1242	16.7%	
South	4398	52.8%	3734	50.3%	
West	1021	12.2%	1219	16.4%	
Hospital size					
Large	6157	73.9%	5659	76.3%	0.085
Medium	1668	20.0%	1248	16.8%	
Small	512	6.1%	512	6.9%	
Surgeon specialty					
General surgeon	4549	54.6%	4346	58.6%	0.081
Colon/rectal surgeon	2784	33.4%	2246	30.3%	
Others/unknown	1004	12.0%	827	11.1%	
Charlson Comorbidity Index scores					
0	3488	41.8%	3055	41.2%	0.053
1–2	2908	34.9%	2476	33.4%	
3–4	965	11.6%	962	13.0%	
5+	976	11.7%	925	12.5%	

^a^After propensity score weighting, the number of patients in the Medtronic EEA^™^ circular stapler with Tri-Staple^™^ technology reflects a weighted sample size

The comparative analysis based on the PSW cohort found that the use of the Ethicon manual circular stapler was not associated with an increased risk of AL within 30 days post-index procedure in comparison to the Medtronic EEA^™^ circular stapler with Tri-Staple^™^ technology (RR, 0.72; 95% CI, 0.52–1.01) (Table 2).

Secondary analyses revealed similar results in patients who did not have a diverting stoma procedure prior to or on the same day as the index procedure. The cumulative incidences (95% CIs) were 7.09% (6.21–8.05%) in the Ethicon manual circular stapler cohort and 7.21% (5.67–9.01%) in the Medtronic EEA^™^ circular stapler with Tri-Staple^™^ technology cohort. After PSW, the RR was 0.77 (0.50–1.18) (Table 2). Similar results were also observed in the sensitivity analysis that only included patients from hospitals with ≥ 30 days continuous enrollment in PHD after the patients’ index procedure (RR, 0.73; 95% CI, 0.52–1.01) (Table 2).

#### Medtronic EEA™ circular stapler with DST™ technology vs. Medtronic EEA™ circular stapler with Tri-Staple™ technology

Observed imbalances between the Medtronic EEA^™^ circular stapler with DST^™^ technology and Tri-Staple^™^ technology cohorts were adequately resolved with PSW (Table 5).

**Table 5:** Patient and hospital characteristics at the index admission after propensity score weighting: Medtronic EEA^™^ circular stapler with DST Series^™^ technology vs. Tri-Staple^™^ technology

	**Medtronic EEA**^**™**^** circular stapler with DST Series**^**™**^** technology**		**Medtronic EEA**^**™**^** circular stapler with Tri-Staple**^**™**^** technology**		**Absolute standardized difference**
	***n***	**%**	***n***	**%**	
*N*	7928		7086^a^		
Sex: male	3733	47.1%	3499	49.4%	0.046
Age					
18–< 45 years	1059	13.4%	930	13.1%	0.034
45–< 65 years	3742	47.2%	3334	47.1%	
65–< 75 years	2046	25.8%	1775	25.0%	
75 years or older	1081	13.6%	1047	14.8%	
Race					
White	6036	76.1%	5730	80.9%	0.125
Black	648	8.2%	531	7.5%	
Others	1105	13.9%	725	10.2%	
Unknown	139	1.8%	100	1.4%	
Procedure year					
2019	3215	40.6%	3005	42.4%	0.090
2020	2052	25.9%	1562	22.0%	
2021	1761	22.2%	1670	23.6%	
2022, through Nov 30 2022	900	11.4%	850	12.0%	
Surgical site					
Rectum	763	9.6%	605	8.5%	0.054
Sigmoid	5043	63.6%	4478	63.2%	
Descending colon	305	3.8%	326	4.6%	
Others	1817	22.9%	1677	23.7%	
Primary diagnosis					
Malignant neoplasms	2358	29.7%	2012	28.4%	0.045
Benign neoplasm	141	1.8%	115	1.6%	
Diverticular disease or diverticulitis	3050	38.5%	2874	40.6%	
Intestinal obstruction	240	3.0%	221	3.1%	
Others	2139	27.0%	1864	26.3%	
Surgical approach					
Open	3328	42.0%	3191	45.0%	0.090
Laparoscopic	2732	34.5%	2144	30.3%	
Robotic assisted	1868	23.6%	1752	24.7%	
Diverting stoma occurred prior to or on the same day as the index procedure	1350	17.0%	1276	18.0%	0.026
Cardiovascular diseases	1147	14.5%	1102	15.5%	0.030
Chronic obstructive pulmonary disease	573	7.2%	582	8.2%	0.037
Coagulation defects	71	0.9%	75	1.1%	0.016
Diabetes	1342	16.9%	1292	18.2%	0.034
Hypertension	4108	51.8%	3688	52.0%	0.005
Immunodeficiency/immunosuppression	135	1.7%	91	1.3%	0.034
Kidney disease	502	6.3%	490	6.9%	0.023
Malnutrition	437	5.5%	265	3.7%	0.084
Obesity	1759	22.2%	1714	24.2%	0.047
Admission type: elective	6780	85.5%	5860	82.7%	0.077
Hospital setting: inpatient	7806	98.5%	7066	99.7%	0.131
Location: urban hospital	7037	88.8%	6180	87.2%	0.048
Teaching hospital (yes)	4515	57.0%	3889	54.9%	0.042
Hospital region					
Midwest	1848	23.3%	1815	25.6%	0.182
Northeast	1777	22.4%	1092	15.4%	
South	3245	40.9%	3218	45.4%	
West	1058	13.3%	961	13.6%	
Hospital size					
Large	6253	78.9%	5424	76.5%	0.119
Medium	1126	14.2%	1283	18.1%	
Small	549	6.9%	379	5.4%	
Surgeon specialty					
General surgeon	4369	55.1%	4257	60.1%	0.123
Colon/rectal surgeon	2484	31.3%	2114	29.8%	
Others/unknown	1075	13.6%	716	10.1%	
Charlson Comorbidity Index scores					
0	3106	39.2%	2833	40.0%	0.052
1–2	2838	35.8%	2512	35.5%	
3–4	917	11.6%	890	12.6%	
5+	1067	13.5%	851	12.0%	

^a^After propensity score weighting, the number of patients in the Medtronic EEA^™^ circular stapler with Tri-Staple^™^ technology reflects a weighted sample size

The comparative analysis based on the PSW cohort revealed no difference in the risk of AL within 30 days post-index procedure comparing the Medtronic EEA^™^ circular stapler with DST^™^ technology to Tri-Staple^™^ technology (RR, 0.75; 95% CI, 0.53–1.06) (Table 3).

Secondary and sensitivity results were consistent with those of the primary analysis (Table 3). Among patients who did not have diverting stoma prior to or on the same day as the index procedure, the RR of AL comparing Medtronic EEA^™^ circular stapler with DST^™^ technology to Tri-Staple^™^ technology was 0.80 (95% CI, 0.51–1.26). Among patients from hospitals with ≥ 30 days continuous enrollment in PHD after the patients’ index procedure, the RR was 0.75 (95% CI, 0.53–1.06).

### Cumulative incidences of AL stratified by patient and provider characteristics

Analyses stratified by key characteristics suggest the risk of AL has remained steady since 2019, when the three-row circular staplers were introduced to the U.S. market (Table 6). The risk of AL in patients who underwent an open procedure was approximately doubled compared to patients who underwent a laparoscopic or robotic-assisted procedure (10.3% vs. 5.9%). Similar AL risk was noted across all 3 device cohorts when stratified by hospital and provider characteristics including hospital size, hospital volume, and surgeon specialty.

**Table 6:** Cumulative incidence (95% confidence interval) of anastomotic leak, by study device and key characteristics

	**Ethicon manual circular staplers**					**Medtronic EEA**^**™**^** circular stapler with DST**^**™**^** technology**							**Medtronic EEA**^**™**^** circular stapler with Tri-Staple**^**™**^** technology**				
	**Number of patients**	**Number of events**		**Cumulative incidence (95% CI)**		**Number of patients**		**Number of events**		**Cumulative incidence (95% CI)**			**Number of patients**			**Number of events**	**Cumulative incidence (95% CI)**
Sex																	
Male	3848	362		9.4% (8.3–10.6%)		3733		330		8.8% (7.8–10.0%)			584			48	8.2% (6.0–11.0%)
Female	4489	287		6.4% (5.5–7.4%)		4195		268		6.4% (5.6–7.2%)			722			59	8.2% (6.3–10.3%)
Age																	
18–< 45 years	1098	79		7.2% (5.9–8.7%)		1059		73		6.9% (5.5–8.6%)			153			12	7.8% (4.1–13.4%)
45–< 65 years	3872	273		7.1% (5.9–8.3%)		3742		259		6.9% (6.0–7.9%)			624			44	7.1% (5.0–9.7%)
65–< 75 years	2132	179		8.4% (7.2–9.7%)		2046		166		8.1% (7.0–9.3%)			353			36	10.2% (6.9–14.5%)
75 years or older	1235	118		9.6% (7.9–11.4%)		1081		100		9.3% (7.3–11.6%)			176			15	8.5% (3.6–16.4%)
Race																	
White	6892	534		7.8% (6.8–8.7%)		6036		425		7.0% (6.3–7.8%)			1131			89	7.9% (6.2–9.8%)
Black	603	63		10.5% (7.7–13.8%)		648		71		11.0% (8.9–13.3%)			90			6	6.7% (3.5–11.3%)
Others	707	45		6.4% (4.7–8.5%)		1105		89		8.1% (6.1–10.4%)			62			7	11.3% (3.3–25.9%)
Unspecified	135	7		5.2% (2.0–10.7%)		139		13		9.4% (4.6–16.6%)			23			NP	NP
Procedure year																	
2019	3317	268		8.1% (6.9–9.4%)		3215		226		7.0% (5.9–8.3%)			293			20	6.8% (4.4–10.0%)
2020	2258	166		7.4% (6.1–8.8%)		2052		160		7.8% (6.6–9.1%)			282			24	8.5% (5.3–12.8%)
2021	1803	138		7.7% (6.3–9.2%)		1761		135		7.7% (6.6–8.9%)			379			33	8.7% (5.9–12.3%)
Through Nov 30 2022	959	77		8.0% (6.0–10.5%)		900		77		8.6% (7.0–10.3%)			352			30	8.5% (5.1–13.2%)
Surgical site based on primary procedure code																	
Rectum	670	54		8.1% (6.0–10.5%)		763		63		8.3% (6.2–10.7%)			166			15	9.0% (5.6–13.7%)
Sigmoid	5395	358		6.6% (5.7–7.7%)		5043		306		6.1% (5.3–6.9%)			838			57	6.8% (5.0–9.1%)
Descending colon	401	33		8.2% (5.6–11.6%)		305		24		7.9% (5.0–11.6%)			55			6	10.9% (5.7–18.4%)
Others	1871	204		10.9% (9.4–12.5%)		1817		205		11.3% (10.0–12.7%)			247			29	11.7% (6.5–19.0%)
Primary diagnosis																	
Malignant neoplasms	2099	175		8.3% (7.0–9.9%)		2358		195		8.3% (7.0–9.7%)			358			24	6.7% (4.1–10.3%)
Benign neoplasm	178	12		6.7% (3.7–11.2%)		141		10		7.1% (3.4–12.8%)			19			NP	NP
Diverticular disease or diverticulitis	3477	224		6.4% (5.4–7.6%)		3050		170		5.6% (4.6–6.7%)			601			38	6.3% (4.5–8.6%)
Intestinal obstruction	243	29		11.9% (8.4–16.3%)		240		27		11.3% (7.1–16.8%)			34			7	20.6% (5.3–46.5%)
Others	2340	209		8.9% (7.8–10.2%)		2139		196		9.2% (7.9–10.5%)			294			37	12.6% (9.3–16.5%)
Surgical approach																	
Open	3566	371		10.4% (9.3–11.6%)		3328		336		10.1% (9.0–11.3%)			409			46	11.3% (8.0–15.3%)
Laparoscopic	2555	142		5.6% (4.1–7.3%)		2732		160		5.9% (4.8–7.0%)			375			22	5.9% (3.3–9.6%)
Robotic assisted	2216	136		6.1% (4.9–7.6%)		1868		102		5.5% (4.4–6.7%)			522			39	7.5% (5.8–9.5%)
Admission type																	
Elective	6894	441		6.4% (5.6–7.3%)		6780		411		6.1% (5.4–6.7%)			1141			84	7.4% (5.9–9.1%)
Urgent/emergency	1443	208		14.4% (12.3–16.7%)		1148		187		16.3% (14.2–18.6%)			165			23	13.9% (8.5–21.2%)
Hospital region																	
Midwest	1208	91		7.5% (5.8–9.6%)		1848		139		7.5% (6.4–8.8%)			328			22	6.7% (2.4–14.5%)
Northeast	1710	106		6.2% (4.2–8.7%)		1777		150		8.4% (7.1–9.9%)			58			12	20.7% (10.9–33.8%)
South	4398	368		8.4% (7.3–9.5%)		3245		233		7.2% (6.0–8.6%)			755			55	7.3% (6.0–8.7%)
West	1021	84		8.2% (6.4–10.3%)		1058		76		7.2% (5.4–9.3%)			165			18	10.9% (7.4–15.4%)
Hospital size																	
Large	6157	465		7.6% (6.5–8.7%)		6253		459		7.3% (6.6–8.2%)			1027			78	7.6% (5.9–9.7%)
Medium	1668	153		9.2% (7.6–11.0%)		1126		90		8.0% (6.3–9.9%)			220			22	10.0% (7.2–13.4%)
Small	512	31		6.1% (4.3–8.2%)		549		49		8.9% (7.0–11.2%)			59			7	11.9% (5.2–22.3%)
Volume of left-sided colorectal surgery per year																	
0–144	1917	160		8.4% (6.9–10.0%)		2161		183		8.5% (7.3–9.8%)			412			37	9.0% (5.4–13.9%)
145–240	2284	190		8.3% (7.2–9.5%)		1203		85		7.1% (5.5–8.9%)			435			35	8.1% (6.2–10.2%)
241–412	1961	158		8.1% (6.1–10.4%)		2496		176		7.1% (5.5–8.8%)			234			13	5.6% (3.1–9.0%)
> 412	2175	141		6.5% (4.7–8.7%)		2068		154		7.5% (6.6–8.4%)			225			22	9.8% (5.4–16.0%)
Surgeon specialty																	
General surgeon	4549	379		8.3% (7.4–9.4%)		4369		344		7.9% (7.1–8.8%)			477			42	8.8% (6.0–12.4%)
Colon/rectal surgeon	2784	170		6.1% (4.9–7.6%)		2484		161		6.5% (5.2–8.0%)			647			46	7.1% (5.0–9.8%)
Others or unspecified	1004	100		10.0% (7.9–12.4%)		1075		93		8.7% (6.5–11.2%)			182			19	10.4% (7.5–14.1%)
Diverting stoma occurred prior to or on the same day as the index procedure																	
Yes	1355	154		11.4% (9.5–13.4%)		1350		147		10.9% (9.2–12.8%)			224			29	13.0% (9.0–17.8%)
No	6982	495		7.1% (6.2–8.1%)		6578		451		6.9% (6.2–7.6%)			1082			78	7.2% (5.7–9.0%)
Cardiovascular diseases																	
Yes	1295	148		11.4% (9.9–13.1%)		1147		112		9.8% (8.0–11.8%)			205			25	12.2% (7.6–18.3%)
No	7042	501		7.1% (6.2–8.1%)		6781		486		7.2% (6.4–8.0%)			1101			82	7.5% (5.8–9.4%)
Chronic obstructive pulmonary disease																	
Yes	599	68		11.4% (8.9–14.2%)		573		53		9.3% (6.8–12.2%)			118			13	11.0% (7.4–15.5%)
No	7738	581		7.5% (6.6–8.5%)		7355		545		7.4% (6.7–8.2%)			1188			94	7.9% (6.2–10.0%)
Coagulation defects																	
Yes	101	11		10.9% (5.5–18.9%)		71		11		15.5% (8.4–25.3%)			13			NP	NP
No	8236	638		7.8% (6.9–8.7%)		7857		587		7.5% (6.8–8.2%)			1293			106	8.2% (6.5–10.1%)
Diabetes																	
Yes	1436	139		9.7% (8.0–11.5%)		1342		119		8.9% (7.2–10.8%)			256			24	9.4% (4.8–16.2%)
No	6901	510		7.4% (6.5–8.4%)		6586		479		7.3% (6.6–8.0%)			1050			83	7.9% (6.3–9.8%)
Hypertension																	
Yes	4407	391		8.9% (7.8–10.0%)		4108		324		7.9% (6.9–9.0%)			723			67	9.3% (7.0–12.0%)
No	3930	258		6.6% (5.5–7.8%)		3820		274		7.2% (6.3–8.1%)			583			40	6.9% (4.7–9.6%)
Immunodeficiency/immunosuppression																	
Yes	115	13		11.3% (5.9–19.0%)		135		15		11.1% (6.8–16.9%)			19			NP	NP
No	8222	636		7.7% (6.9–8.7%)		7793		583		7.5% (6.8–8.2%)			1287			106	8.2% (6.6–10.1%)
Kidney disease																	
Yes	601	65		10.8% (8.4–13.7%)		502		42		8.4% (6.2–11.0%)			91			10	11.0% (5.4–19.4%)
No	7736	584		7.6% (6.7–8.5%)		7426		556		7.5% (6.8–8.2%)			1215			97	8.0% (6.4–9.9%)
Malnutrition																	
Yes	397	83		20.9% (16.9–25.4%)		437		63		14.4% (11.2–18.1%)			59			12	20.3% (12.1–31.0%)
No	7940	566		7.1% (6.3–8.1%)		7491		535		7.1% (6.5–7.9%)			1247			95	7.6% (6.0–9.6%)
Obesity																	
Yes	1885	186		9.9% (8.4–11.6%)		1759		156		8.9% (7.5–10.4%)			334			28	8.4% (5.6–12.0%)
No	6452	463		7.2% (6.3–8.1%)		6169		442		7.2% (6.5–7.9%)			972			79	8.1% (6.3–10.4%)

*CI* confidence interval, *NP* not presented (when fewer than 30 patients were eligible for the analysis, data is not presented)

## Discussion

We present the first large U.S.-based cohort study of left-sided colorectal resection for AL risk based on circular stapler device used, specifically comparing two- and three-row devices. Both the Ethicon and Medtronic two-row staplers had similar AL risks as the three-row stapler before and after PSW. Similar results were found for patients who did not have a diverting stoma prior to or on the same day as a left-sided colorectal surgery during the index admission.

The observed cumulative incidences of AL in this study for a two-row manual circular stapler are consistent with those reported in an observational study conducted using this same data source [23], three single-center studies [10, 17, 18] and one multicenter observational study [21] with a head-to-head comparison between two-row and three-row circular staplers.

In contrast, the estimated cumulative incidences of AL in patient procedures performed using a three-row circular stapler were below 3% in four previously published studies [10, 17, 18, 21], lower than the present study [8.2% (6.6–10.1%)], even among those who underwent a minimally invasive [5.9% (3.3–9.6%)] or robotic-assisted surgery [7.5% (5.8–9.5%)]. This difference could be partially explained by differences in outcome ascertainment. A gold standard for diagnosing AL is currently lacking. The diagnosis of an AL generally requires patient’s global clinical assessment, adjunctive laboratory data, and radiological assessment, and the decision of a diagnosis sometimes can be different depending on physicians’ clinical judgment [28, 29]. In a retrospective observational study based on the review of patient’s medical records, without an adjudication process in place, investigators’ knowledge of exposure status and study hypothesis (i.e., unblinded outcome classification) could have resulted in potential for differential misclassification of AL, particularly for less severe AL. In the Quero et al. study, less severe AL cases (i.e., ISREC grade A or B) accounted for only 1/3 of AL identified in the three-row cohort while 74% of ALs identified in the two-row population were less severe^10^. This could lead to an underestimation of AL risk in patient procedures using a three-row manual circular stapler in these four published studies based on review of medical records. In contrast, the ascertainment of AL in the present study was based on the identification of existing diagnosis and/or procedural codes readily available in the PHD, which were collected and maintained for billing or record-keeping purposes prior to the conduct of this study and independent of the study hypothesis.

Observational studies using real-world data such as the PHD have both strengths and limitations. Relative to four recently published studies [10, 17, 18, 21], the key strengths of this study are the large and regionally diverse sample of U.S. patients as well as the rigorous approach to addressing confounding, which improves the comparability between the two-row and three-row manual circular stapler groups. As with other studies examining colorectal AL, there are limitations related to the definition and diagnostic criteria, as there is no specific code for AL in the ICD-10-CM taxonomy and surrogate diagnoses must be used [29]. Misclassification bias would result if study patients were not categorized correctly with regard to outcome; since the AL definition was applied equally across all the three cohorts, this would be nondifferential and the impact could be a bias towards the null. PHD is not a longitudinal patient database; rather, it is a longitudinal hospital database for the duration of continuous participation in PHD from each institution. Longitudinal data are available only for follow-up encounters within the same hospital where the index procedure was performed. This could theoretically lead to underreporting of AL risks if patients were discharged and presented with new AL to a different hospital. However, we expect this underestimation to be low, as patients with surgical complications that occur within 30 days of a surgical procedure would be likely to return to the same hospital where they received the surgical procedure. Should underestimation occur, this would probably be nondifferential among study cohorts, as patient decisions to return to the same hospital unlikely depend on device used. Furthermore, a sensitivity analysis including patients from hospitals with ≥ 30-day continuous enrollment in PHD after the patients’ index procedure produced results that were consistent with those of the primary analysis suggesting that misclassification of outcome resulting from loss of institution continuous enrollment in the PHD is unlikely.

## Conclusion

In conclusion, in the analysis of a large cohort of patients undergoing a left-sided colorectal surgery from a U.S. hospital database, the risk of AL observed with manual two-row circular staplers is similar to that seen with three-row devices. This study affirms the safety of manual two-row circular staplers in colorectal anastomosis.

## Data Availability

Data used in this analysis were extracted from the Premier Healthcare Database (owned by Premier Inc., Charlotte, NC, USA) on February 27, 2023.
